# Integrating toda embroidery with pre-consumer textile waste: A sustainable approach to fashion production and cultural preservation

**DOI:** 10.1016/j.mex.2025.103339

**Published:** 2025-04-28

**Authors:** Ramya Arivanantham, Vineeth Radhakrishnan

**Affiliations:** Vellore Institute of Technology, Chennai Campus, India

**Keywords:** Toda embroidery, Sustainable fashion, Upcycling, Textile waste management, Cultural preservation, Sustainable Textile Repair through Indigenous Embroidery (STRIE) Method

## Abstract

This research aims to develop a systematic approach for reducing pre-consumer textile waste by integrating traditional Toda embroidery techniques into garment repair. This study focuses on promoting environmental sustainability while preserving indigenous cultural heritage. Drawing parallels with the Japanese *Kintsugi* concept, which transforms broken ceramics into aesthetic enhancements, this study proposes a structured method to elevate defective textiles through Indigenous craftsmanship. The Sustainable Textile Repair through Indigenous Embroidery (STRIE) Method consists of four phases, i) Defect Assessment ii) Artisan Engagement iii) Repair Execution and iv) Quality Assurance. This framework extends garment lifespans, reduces environmental impact and creates sustainable economic opportunities for Toda artisans. The STRIE Method presents a scalable framework for sustainable textile repair, strategically aligning with four United Nations Sustainable Development Goals (SDGs 8, 11, 12, 13). This innovative strategy facilitates the conversion of textile waste into garments that carry cultural significance while promoting environmental sustainability. It adeptly fuses traditional craftsmanship with modern sustainable production techniques, creating a model that enhances both the ecological and cultural value of textile products.

• Introduces a structured integration of traditional craftsmanship into modern repair.
• Combines digital defect assessment with artisan training and quality assurance.
• Reduces textile waste, preserves cultural heritage and supports sustainable livelihoods.

## Graphical abstract

**Figure:**
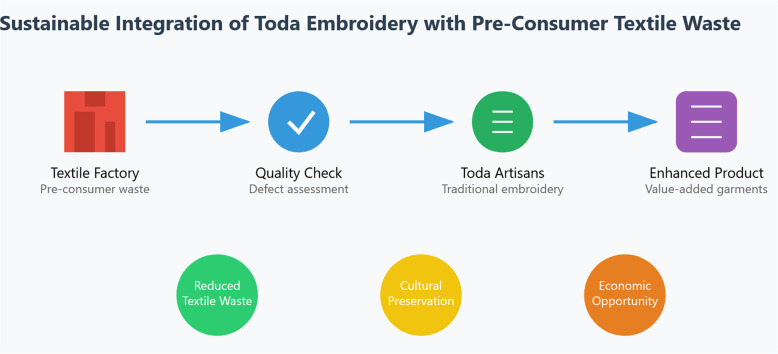

Specifications table

**Table:** 

Subject area:	Environmental Science
More specific subject area:	Fashion Studies, Sustainable Textile Production
Name of your method:	Sustainable Textile Repair through Indigenous Embroidery (STRIE) Method
Name and reference of original method:	Integration of traditional craft with modern fashion production, as demonstrated in: Ahosanul Karim et al. [1]. “Transformative adoption of traditional ‘kantha’ embroidery to modern fashion design through ‘khadi’ fabric.” American Journal of Art and Design, 6(1), 6. https://doi.org/10.11648/j.ajad.20210601.12
Resource availability:	• De Castro, O. (2022). Loved clothes last: How the joy of rewearing and repairing your clothes can be a revolutionary act. Penguin Life. • Sharma, G., & Bhagat, S. (2018). Revival of Toda embroidery-needlecraft of Nilgiris. Jurnal Sosioteknologi, 17(1), 1–13. https://doi.org/10.5614/sostek.itbj.2018.17.1.1

## Background

The global textile industry is a major contributor to environmental degradation, largely due to its extensive production processes. A comprehensive industry analysis by Tripathi et al. [2] indicates that India alone generates approximately 7793 kilotons of textile waste annually, constituting 8.5 % of global textile waste production. Within this framework, the Tirupur textile cluster in Tamil Nadu responsible for 40 % of India’s knitwear exports—produces a significant volume of pre-consumer waste, much of which remains underutilized [3]. Simultaneously, traditional artisanal practices such as Toda embroidery face an existential threat due to the encroachment of modern market forces that increasingly render them obsolete. This indigenous craft, deeply embedded in cultural and historical traditions, presents a compelling avenue for sustainable innovation in textile waste management. The proposed methodology integrates textile waste repair with Toda embroidery techniques, offering a dual-pronged approach that not only mitigates environmental impact but also enriches the cultural value of repurposed garments. Moreover, this initiative fosters economic resilience among artisans by creating new market opportunities, thereby ensuring the continuity of heritage craftsmanship in contemporary sustainable fashion practices.

As shown in Table 1, pre-consumer waste constitutes 42 % of India’s total textile waste, with half of this amount containing repairable defects that could potentially be salvaged through interventions like the STRIE Method.

**Table 1:** Textile Waste Statistics in India.

Category	Quantity	Percentage	Source
Annual Textile Waste	7793 kilotons	60 %	Tripathi et al. [2]
Pre-consumer Waste	5195 kilotons	42 %	Mohan et al. [3]
Repairable Defects	<3896 kilotons	<50 %	Mohan et al. [3]
Recycled	3117 kilotons	25 %	Solanki [4]

## Textile waste and overproduction

The textile industry constitutes a pivotal sector within the global economy, particularly in leading Asian nations. However, the escalating scale of textile production has precipitated severe environmental repercussions, including adverse impacts on climate, depletion of water resources and excessive energy consumption [5,6]. Over the past 15 years, clothing production has doubled, yet consumer behaviors often reflect a disregard for sustainability, with garments frequently remaining unworn or being discarded without consideration of their ecological footprint [7]. Historically, clothing has served as a fundamental marker of identity and professional status [8]; however, the luxury fashion sector has contributed to the commodification of fashion as a transient, superficial pursuit, largely driven by media-driven consumerist narratives. While discourse on textile waste predominantly centers on the proliferation of fast fashion and premature garment disposal, an equally pressing issue lies within the production stage. Large-scale textile manufacturing generates substantial pre-consumer industrial waste due to weaving and printing defects, material imperfections and contamination from oils and dyes [9].

Modern societies continue to grapple with the environmental and social ramifications of prevailing production and consumption models, underscoring the urgency of a sustainability transition [10]. De Castro [7], in *Loved Clothes Last: How the Joy of Rewearing and Repairing Your Clothes Can be a Revolutionary Act*, advocates for a cultural shift toward mending and re-wearing garments as a means of fostering environmental consciousness and emotional connection to clothing.

Upcycling is defined as “a process in which products and materials that are no longer in use, or are about to be disposed of, are instead repurposed, repaired, upgraded and remanufactured in a way that increases their value” ([11], p. 1), differs fundamentally from recycling, which often degrades material quality. Upcycling preserves and enhances the inherent value of textiles, positioning itself as a viable response to resource depletion and escalating waste generation [12]. Beyond its environmental benefits, upcycling has demonstrated the potential in fostering economic opportunities, stimulating entrepreneurship and promoting sustainable consumption behaviors. While existing scholarship has primarily examined upcycling within the post-consumer context [13,14], emerging research highlights the environmental advantages of repurposing pre-consumer textile waste within the fashion supply chain [2,15]. Statistical analyses reveal that out of the 100 billion garments produced annually, approximately 92 million tons of textiles are discarded during the production and pre-consumer phases, ultimately contributing to landfill overflow [16]. Industry reports from 2018 to 2019 identify Gujarat, Karnataka and Tamil Nadu as India’s primary textile and apparel hubs, with the Tirupur belt in Tamil Nadu playing a crucial role in cotton garment manufacturing, driven by regional climatic conditions and sustained consumer demand [3].

Upcycling not only facilitates material repurposing but also fosters a deeper engagement with temporality, transformation and cultural heritage. Through the reinvention of discarded textiles, individuals develop a heightened awareness of material properties, artistic traditions and historical narratives, thereby making meaningful contributions to the intersection of fashion, sustainability and community-driven economic initiatives. This study positions upcycling as a critical response to the ethical dilemmas within the contemporary textile industry, advocating for an expanded definition that transcends conventional manufacturing frameworks centered on corporate profit. Instead, upcycling should be reimagined as an artistic and historically informed practice that valorizes Indigenous craft traditions, fosters cultural continuity and contributes to community resilience within a sustainable fashion paradigm.

## Method details

This study introduces a pioneering methodological approach for the production phase, mitigating the disposal of minimally defective garments in landfills while bridging sustainability and cultural heritage. Central to this framework is the global dissemination of Toda embroidery, a traditional Indian art form known as *puhkoor*, historically practiced on *puthukuli*, the intricately embroidered shawls of the Toda people. The Toda, a small Indigenous community residing in the Nilgiri Mountains of Tamil Nadu, are speakers of a Dravidian language and sustain their livelihoods primarily through buffalo dairy farming and intricate needlework. Their embroidery technique is highly distinctive, characterized by the exclusive use of black and red woolen threads, worked with a long needle. Unlike conventional embroidery methods, Toda artisans forego embroidery hoops, relying instead on precise finger-based counting and weaving techniques. This results in a fully reversible textile, allowing both sides of the fabric to be displayed. Toda women, recognized for their extraordinary craftsmanship, directly create complex patterns onto fabric without preliminary sketching, drawing inspiration from natural elements such as wildflowers, mountain landscapes, rabbit ears, the sun, the moon and valleys [17]. The *puthukuli* shawl, embroidered exclusively by Toda women, holds profound cultural significance, serving as a ceremonial garment worn by both men and women, particularly during weddings and other pivotal social occasions. Its motifs symbolize prosperity, fertility and security, rendering it integral to the community’s cultural identity [18]. In recent years, Toda embroidery has been adapted to contemporary applications, extending beyond traditional shawls to include cushion covers, mobile pouches, handbags, table runners and mats [18,19] (Fig. 2).

Drawing upon established scholarship in sustainable fashion, Indigenous craft preservation and textile waste management, this study presents an innovative paradigm for garment repair. Prior research has demonstrated the potential of integrating traditional embroidery techniques with sustainable textile practices. For instance, Ahosanul Karim et al. [1] examined the revitalization of Kantha embroidery through its fusion with Khadi fabric, while Brownlow et al. [20] documented the global market integration of Andean weaving traditions. Existing literature also highlights the vulnerability of Toda embroidery, which remains confined to the domestic market and faces the risk of decline. Ensuring its long-term viability necessitates a dual commitment to environmental sustainability and the socio-cultural preservation of this endangered art form.

This paper advances a conceptual framework that incorporates Toda embroidery techniques into pre-consumer textile waste repair, drawing theoretical parallels with the Japanese philosophy of *Kintsugi*, wherein broken pottery is repaired with gold, transforming fractures into aesthetic and symbolic enhancements [22]. By applying this principle to garment restoration, the study seeks to advance sustainable fashion by elevating discarded textiles through Indigenous craftsmanship. This approach presents a replicable model for reducing industrial textile waste while simultaneously enriching cultural heritage and aesthetic value. Moreover, the integration of Tirupur’s cotton industry into this initiative offers a strategic pathway for amplifying sustainable fashion practices on a global scale, merging eco-conscious textile production with Indigenous artisanal traditions to establish an impactful paradigm in contemporary fashion.

Ultimately, this study calls for a redefinition of upcycling within a material-culture framework, shifting the prevailing focus from economic valuation to the artistic reclamation of devalued materials. By fostering deeper emotional and cultural connections between individuals and textiles, the study proposes an alternative, heritage-driven model of sustainability. This theoretical reorientation is operationalized through the STRIE Method, which introduces three key innovations in sustainable textile management:

1. A standardized protocol for classifying industrial textile defects to determine suitability for craft-based intervention.
2. A reproducible framework for adapting Toda embroidery motifs while ensuring cultural authenticity and integrity.
3. A set of quantifiable quality control parameters that safeguard both the technical durability and the cultural preservation of embroidered textiles.

**Fig. 1:**
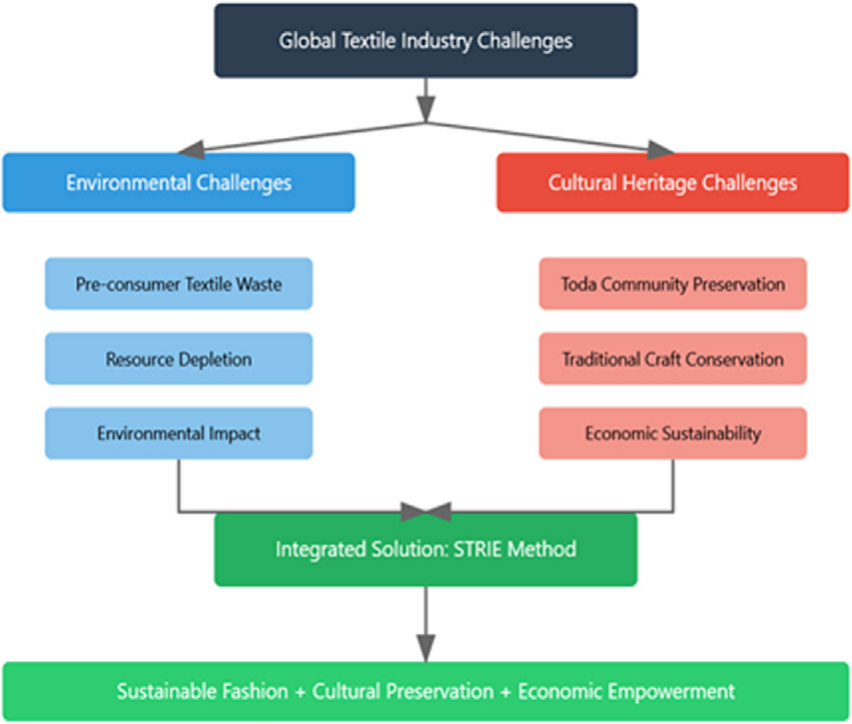
illustrates the integration of environmental and cultural challenges in the textile industry through the Sustainable Textile Repair through Indigenous Embroidery (STRIE) Method.

By embedding Indigenous craftsmanship within sustainable textile innovation, this framework not only mitigates industrial waste but also fosters a dynamic interplay between tradition and contemporary sustainable fashion, ensuring the longevity and global visibility of Toda embroidery (Fig. 1).

**Fig. 2:**
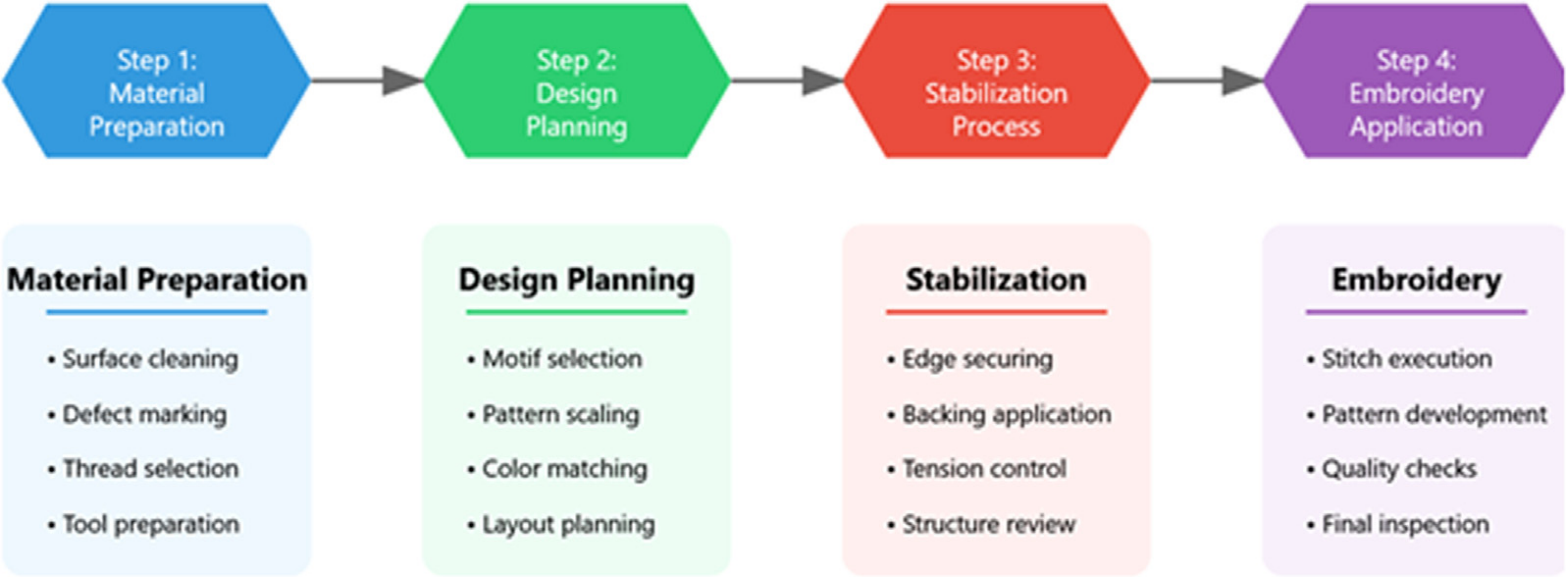
illustrates the systematic integration of traditional Toda embroidery with modern repair techniques. Each step incorporates quality control measures and documentation requirements to ensure consistency and reproducibility.

Integrating Toda embroidery into the remediation of garments discarded due to minor textile imperfections within the production phase presents both cultural and environmental imperatives. This approach holds significant potential for advancing sustainability while safeguarding Indigenous artisanal traditions. Recognizing the distinctiveness of Toda embroidery, the tribal representatives were granted Geographical Indication (GI) status to prevent the proliferation of low-quality imitations, thereby securing the authenticity and economic viability of this heritage craft [23]. Despite these protections, the broader textile industry in India continues to grapple with inefficiencies in textile waste management, with <50 % of discarded materials being repaired and repurposed. While certain forms of waste, such as fabric deadstock, apparel overproduction and pure white cotton remnants, are effectively recycled, substantial quantities of solid-colored cotton remain underutilized due to technological constraints in recycling processes [3].

Through an extensive analysis of secondary sources encompassing sustainable fashion methodologies, Indigenous craft documentation and textile waste management strategies, this paper constructs a theoretical framework for embedding Toda embroidery within pre-consumer textile repair. Drawing from interdisciplinary scholarship on traditional craft preservation, sustainable design and Indigenous knowledge systems, the research critically examines their intersection to contribute to the evolving discourse on sustainable fashion. By positioning Indigenous embroidery as a viable intervention in modern industrial practices, this study underscores the broader potential of heritage crafts in reshaping contemporary sustainability initiatives.

This framework challenges the prevailing commercialized notion of upcycling by reconceptualizing it as an act of slow care work a process that foregrounds the historical and material significance of textiles rather than reducing them to mere economic commodities. By decentering a market-driven approach, the study advocates for a more nuanced engagement with sustainable design, one that re-historicizes discarded materials and situates them within interconnected human and non-human narratives. This paradigm shift not only mitigates environmental impact but also fosters an integrated, systemic approach to sustainable textile intervention, structured across four primary phases:

• Defect Assessment: Identifying and categorizing textile imperfections based on their suitability for embroidery-based repair.
• Artisan Engagement: Collaborating with Toda craftspeople to ensure cultural authenticity in motif selection and technique application.
• Repair Execution: Implementing embroidery interventions that align with the structural and aesthetic needs of defective textiles.
• Quality Assurance: Establishing evaluation metrics that uphold both technical durability and cultural integrity in the repaired garments.

While prior research has investigated sustainable textile waste management [2] and the preservation of traditional crafts [18], this study introduces an innovative classification system that systematically maps specific textile defects to appropriate Toda embroidery motifs and techniques. This classification framework enables a standardized, replicable method in which distinct defect categories including surface irregularities, color inconsistencies, structural flaws, print distortions and oil stains are systematically paired with corresponding Toda embroidery interventions. By integrating a multi-dimensional assessment model that encompasses environmental, economic and cultural impact analyses, this research addresses a critical gap in existing methodologies, which often prioritize either ecological sustainability or socioeconomic outcomes without incorporating cultural preservation as a fundamental evaluative criterion.

The novelty of the STRIE Method lies in its structured application of Indigenous embroidery techniques for pre-consumer textile repair, introducing three key innovations: i) a standardized protocol for classifying and preparing industrial textile defects for embroidery-based remediation ii) a reproducible framework for adapting Toda embroidery motifs to contemporary textile repair while ensuring cultural fidelity and iii) quantifiable quality control parameters that safeguard both the technical durability and the heritage value of embroidered garments. This research engages with the inquiry, how can traditional Toda embroidery be systematically integrated into contemporary textile production to simultaneously mitigate industrial waste, preserve cultural heritage and generate sustainable livelihood opportunities for Indigenous artisans? By advancing a framework that intertwines cultural sustainability with environmental responsibility, this study redefines the role of traditional craftsmanship in contemporary fashion, offering a transformative model for ethical and sustainable textile practices.

## Phase 1: defective garment assessment protocol

The initial phase entails a rigorous and systematic evaluation of pre-consumer textile waste to determine its viability for repair through the application of Toda embroidery. Adopting the quality assessment framework delineated by De Silva [9], this process employs a set of predefined criteria to assess garment suitability for artisanal intervention, ensuring both technical feasibility and aesthetic coherence.

### Required materials

• Digital caliper (precision: ±0.02 mm) for precise defect measurement
• Color calibration card (X-Rite Color Checker) to ensure accurate color assessment
• Documentation form for systematic data recording
• Digital camera (minimum 12MP resolution) for high-fidelity defect imaging

## Procedure

1. Position the garment on a neutral grey surface (Pantone 17–0000 TCX) under standardized lighting conditions (D65, 6500 K) to ensure uniform color representation, which is a standard viewing of color assessment.
2. Capture high-resolution images of the defect, incorporating the color calibration card within the frame to facilitate accurate color reproduction.
3. Utilize the digital caliper to obtain precise measurements of the defect dimensions, ensuring consistency in assessment.
4. Document the defect characteristics using standardized terminology as outlined in the Textile Defect Glossary [21], ensuring terminological precision and methodological consistency (Table 2).

**Table 2:** Defect Assessment Criteria for STRIE Method Implementation.

Defect Category	Acceptable Parameters	Intervention Potential
Surface Imperfections	≤ 5cm² area	High
Color Variations	Minor shade differences	Medium
Structural Issues	Small tears < 3cm	Medium
Print Defects	Misalignment < 2cm	High
Oil Stains	Diameter < 4cm	Medium

Source: Adapted from “Textile Quality Assessment Guidelines” [21].

## Phase 2: Toda artisan integration protocol

The efficacy of the STRIE (Sustainable Textile Repair through Indigenous Embroidery) method is contingent upon the meaningful engagement of Toda artisans, ensuring the preservation of their traditional embroidery techniques while aligning with contemporary production standards. Drawing upon the community engagement framework proposed by Sharma and Bhagat [18], this study establishes a structured protocol for artisan integration, balancing cultural heritage conservation with industrial adaptability.

### Skill assessment and training program

A comprehensive skill development framework was designed in accordance with the National Handicrafts Development Programme (NHDP) [24], fostering the seamless integration of Toda embroidery within modern garment repair processes. The training program encompasses three core components:

#### Technical skill enhancement

• Adaptation of traditional stitches to accommodate diverse fabric compositions
• Modern fabric handling techniques to ensure compatibility with contemporary textiles
• Implementation of quality control measures to maintain consistency across production

#### Production integration

• Workflow management strategies to optimize efficiency within production cycles
• Standardized documentation practices for ensuring traceability and quality assurance
• Timeline adherence protocols to facilitate integration into structured supply chains

### Design adaptation (3–4 days)

The design adaptation phase ensures the seamless incorporation of Toda embroidery into textile repair interventions. This process involves:

#### Motif selection

Artisans select the appropriate Toda motif based on:

• Defect dimensions (size and shape)
• Garment characteristics (color, texture and material composition)
• Target market segment (aesthetic and commercial considerations)

#### Pattern scaling

Traditional Toda motifs are adapted to suit the size of textile defects through a structured pattern application framework:

• Small defects: Single-motif embellishment for localized repairs
• Medium defects: Clustered motif arrangements for enhanced visual integration
• Large defects: Extended pattern sequences ensuring cohesive design continuity

By merging traditional craftsmanship with structured training and adaptation methodologies, this protocol establishes a sustainable, scalable model for the reintegration of Toda artisans into contemporary fashion production, ensuring both economic viability and cultural preservation.

## Phase 3: repair execution framework

The repair execution follows a standardized process that combines traditional Toda embroidery techniques with modern textile repair practices. This integration builds upon sustainable repair methodologies documented by Tripathi et al. [2] and traditional craft preservation approaches discussed by Varghese et al. [19].

### Validation and repository structure

To uphold authenticity and maintain historical continuity, each repair is subjected to a rigorous validation process:

• Expert panel review, comprising a minimum of three senior Toda artisans
• Comparative photographic analysis, cross-referencing with archival Toda embroidery collections

To further enhance accessibility and facilitate scholarly research, the repair records are integrated into a structured digital repository featuring:

• Unique repair identification codes (format: YY-MM-DD-ID-Type)
• A centralized digital archive with secure cloud-based backup
• Metadata tagging for streamlined searchability and retrieval
• Knowledge transfer documentation, serving as an educational resource for artisan training and academic study

By embedding cultural integrity, methodological rigor and digital accessibility into the documentation process, this framework not only preserves Toda embroidery traditions but also fosters their sustainable integration into contemporary textile practices.

### Validation and impact assessment

Building on methodologies established by Roos et al. [25] and Muthu [21], this study developed a comprehensive impact assessment framework that quantifies the environmental benefits of the STRIE Method compared to conventional disposal practices.

As demonstrated in Table 3, implementation of the STRIE Method substantially reduces environmental impacts across key metrics when compared to conventional disposal practices, with particularly significant reductions in textile waste generation.

**Table 3:** Environmental Impact Metrics with Secondary Source Validation.

Parameter	Conventional Disposal Range	STRIE Method Solution	Source
Textile Waste Generated	Approximately 85 % of all textiles are discarded each year, contributing significantly to environmental pollution.	Toda embroidery encourages upcycling and garment repair, preventing premature disposal and reducing landfill waste.	[[25], [26]]
Carbon Footprint	The textile industry contributes up to 8 % of global carbon emissions due to energy-intensive processes.	Using Toda embroidery extends the lifespan of garments, reducing the demand for mass production, energy-intensive manufacturing and transportation emissions.	[27]
Water Usage	Textile production accounts for about 20 % of global clean water pollution from dyeing and finishing products.	Toda embroidery promotes handcrafting techniques, reducing reliance on water-intensive industrial dyeing and processing methods.	[28,29]

## Phase 4: quality assurance and documentation

The quality assurance protocol follows standardized metrics adapted from both traditional textile manufacturing and artisanal craftsmanship. Building on the quality assessment framework developed by Mohan et al. [3], this study implemented a comprehensive evaluation system that ensures both technical and cultural integrity.

Table 4, outlines the rigorous quality metrics implemented to ensure repaired textiles meet both technical standards and cultural authenticity requirements.

**Table 4:** Quality Control Parameters for STRIE Method.

Stitch Density	8–12 per cm	Digital measurement
Color Match	Original fabric	Spectrophotometer
Tensile Strength	Original fabric	ASTM D5034
Pattern Integrity	Traditional motifs	Visual inspection
Reversibility	Both sides usable	Visual inspection

### Comprehensive documentation and cultural validation framework

In alignment with the documentation framework outlined by Ahosanul Karim et al. [1], each repair intervention undergoes meticulous documentation to ensure transparency, reproducibility and cultural preservation. The documentation process is structured into three key components:

#### Digital documentation

• High-resolution digital photography capturing:
• Pre-repair garment condition
• Step-by-step repair process
• Final restoration outcomes
• Microscopic analysis where applicable

#### Technical documentation

• Detailed material specifications, including fabric composition and dye properties
• Step-by-step intervention procedures, ensuring process standardization
• Quality assessment metrics, incorporating textile durability tests
• Artisan annotations, providing insights into repair methodology and execution

#### Cultural documentation

• Identification of traditional Toda motifs, ensuring design authenticity
• Analysis of pattern significance, contextualizing motifs within Toda heritage
• Justification for adaptation, outlining modifications made for repair feasibility
• Preservation of cultural narratives, ensuring the respectful application of traditional aesthetics

This multi-tiered documentation strategy not only enhances the scalability and reproducibility of the repair methodology but also safeguards cultural knowledge transmission to future practitioners and scholars.

### Cultural authenticity assessment

Following the cultural authenticity framework proposed by Nair et al. [17], each repair intervention is rigorously evaluated based on a standardized rubric to ensure fidelity to Toda embroidery traditions.

#### Motif fidelity (Scale 1–5)

• Preservation of traditional proportions in motif execution
• Adherence to authentic color schemes (restricted to Toda embroidery’s signature black and red)
• Expert recognition of patterns as consistent with Toda heritage

#### Contextual appropriateness (Scale 1–5)

• Alignment between the motif and garment type/function
• Ethical considerations in the adaptation of sacred symbols
• Maintenance of cultural storytelling through embroidery placement

#### Technical execution (Scale 1–5)

• Application of traditional Toda stitching techniques
• Consistency with historical precedents, verified against archival references
• Reversibility assessment, ensuring that the embroidery does not permanently alter the garment’s structural integrity

**Fig. 3:**
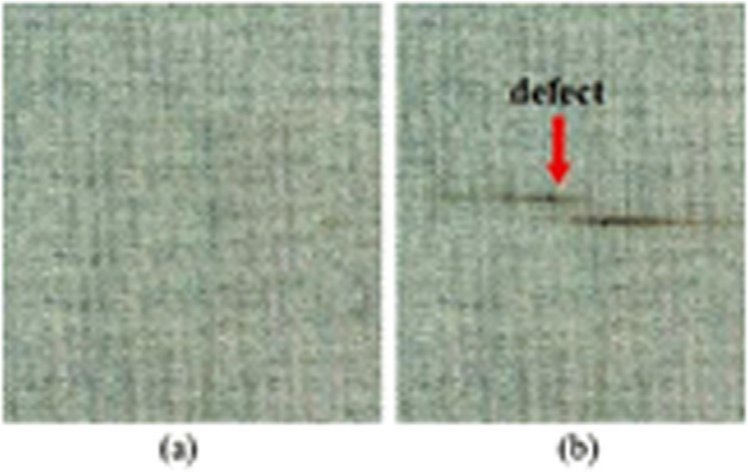
Minor color defect material, sourced from Common fabric defects and its causes.

**Fig. 4:**
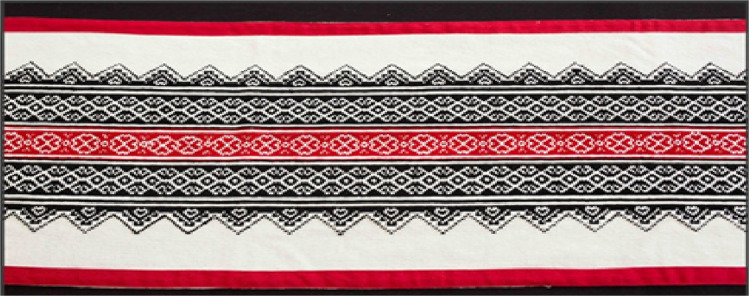
Toda embroidery, sourced from Toda embroidery digitization.

**Fig. 5:**
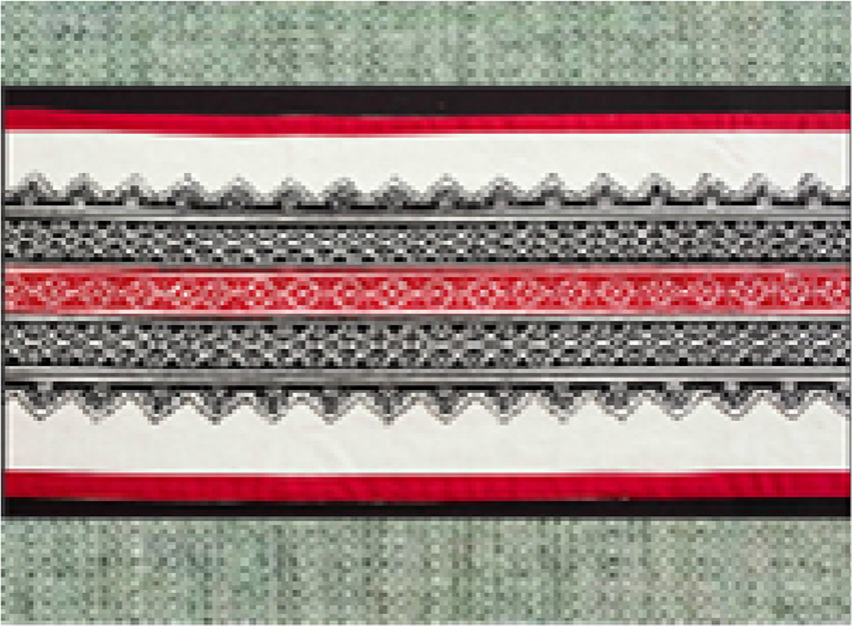
Repaired garment with Toda embroidery (Sample Model).

Fig. 3 displays defective material, which has a minor stain and is categorized as fabric deadstock; this material has been removed from the production stage. Fig. 4 features a Toda embroidery shawl adorned with traditional Toda motifs. Fig. 5 showcases a repaired garment. The method proposed in the paper suggests using Toda embroidery as a solution to reduce pre-consumer textile waste and to preserve the cultural heritage of the Nilgiris.

## Replication protocol for the strie method: A knowledge transfer framework

To facilitate the scalability and adaptability of the STRIE Method across diverse contexts and organizational structures, this study has developed a systematic replication protocol informed by the knowledge transfer frameworks of Ahosanul Karim et al. [1] and Tripathi et al. [2]. This protocol ensures methodological integrity while allowing for context-sensitive modifications, thereby fostering sustainable and culturally responsive implementation.

## Assessment and adaptation phase (2–4 weeks)

• Comprehensive analysis of local textile waste streams, identifying materials suitable for repair and upcycling
• Mapping of indigenous craft opportunities, assessing the feasibility of integrating regional artisanal traditions into the repair methodology
• Contextual adaptation of methodology, ensuring alignment with local textile ecosystems and socio-cultural dynamics

## Capacity building phase (4–8 weeks)

• Strategic artisan identification and engagement, fostering equitable collaboration with traditional craft communities
• Implementation of a structured training program, equipping artisans with the necessary technical skills to integrate traditional embroidery into textile repair
• Establishment of a rigorous quality assurance system, ensuring consistency and authenticity in repair interventions

## Pilot implementation phase (8–12 weeks)

• Execution of small-scale trial production, assessing the practical efficacy of the adapted methodology
• Iterative refinement of processes, incorporating feedback loops to enhance technical precision and cultural fidelity
• Comprehensive documentation and knowledge capture, preserving process insights for future scalability

## Scaling and integration phase (12+ weeks)

• Gradual expansion of production capacity, ensuring sustainable growth without compromising craftsmanship quality
• Strategic supply chain integration, optimizing material procurement and distribution networks
• Implementation of a continuous improvement framework, facilitating ongoing methodological refinement and adaptation to evolving industry needs

This replication framework ensures that the core innovations of the STRIE Method including its emphasis on sustainability, cultural heritage preservation and artisanal empowerment can be effectively transferred across diverse settings. By harmonizing methodological rigor with localized adaptations, this protocol fosters a globally scalable yet culturally inclusive approach to textile repair and heritage craft revitalization.

## Cultural preservation and economic opportunities

This methodological approach uniquely integrates traditional Toda embroidery with textile repair, offering a replicable framework for mitigating pre-consumer textile waste while simultaneously enhancing garments with cultural significance and aesthetic value. By embedding Indigenous craftsmanship within contemporary fashion practices, this model not only elevates the desirability and marketability of repaired garments but also fosters deeper appreciation for heritage textiles. However, traditional Toda embroidery faces existential threats due to market volatility, declining sales and unauthorized design replication. On 17 July 2019, *The Hindu* reported that these challenges endanger the livelihoods of over 300 Toda women artisans in the Nilgiris, underscoring the urgent need for sustainable economic interventions to support this craft [30].

The environmental ramifications of conventional textile manufacturing are profound, encompassing resource-intensive production processes that generate substantial waste and emissions. These industrial activities contribute significantly to greenhouse gas emissions, climate change acceleration, fossil fuel depletion, water pollution, toxicity proliferation and ecosystem degradation at local, regional and global scales [21]. In contrast, Toda embroidery operates within an eco-conscious paradigm, utilizing locally cultivated cotton and natural dyes that align with sustainable production principles. Integrating Toda artisans into contemporary fashion supply chains presents an opportunity to both safeguard their artistic heritage and establish equitable, sustainable income streams. This collaborative approach fosters a symbiotic relationship between ancestral textile traditions and modern manufacturing, cultivating a production model that foregrounds both environmental responsibility and cultural preservation.

To operationalize and scale this initiative, forging strategic partnerships among textile manufacturers, fashion brands and non-governmental organizations (NGOs) is essential. These collaborations would facilitate the integration of repaired garments into global supply chains, leveraging the dual narratives of cultural heritage and environmental sustainability to enhance consumer engagement. This initiative directly aligns with several United Nations Sustainable Development Goals (SDGs), particularly SDG 8 (Decent Work and Economic Growth) this initiative fosters sustainable livelihoods for over 300 Toda women artisans, addressing the economic marginalization they face by securing fair wages and stable employment. By integrating these craftspeople into sustainable supply chains, the model advances inclusive economic development while simultaneously safeguarding intangible cultural heritage, SDG 11 (Sustainable Cities and Communities) this initiative contributes to the preservation and revitalization of intangible cultural heritage, ensuring the intergenerational transmission of Toda embroidery traditions. By embedding these craft practices within sustainable fashion ecosystems, the model not only safeguards an endangered artisanal legacy but also fosters cultural resilience within Indigenous communities, SDG 12 (Responsible Production and Consumption) this intervention directly mitigates textile waste by extending garment lifespans through culturally embedded repair practices, thereby challenging the disposability-driven logic of fast fashion. By prioritizing resource efficiency and leveraging locally sourced cotton and natural dyes, the approach minimizes the ecological footprint associated with conventional textile manufacturing and SDG 13 (Climate Action) this initiative promotes garment repair over new production, this framework significantly reduces greenhouse gas emissions, energy consumption and material extraction—key contributors to climate change. Extending textile longevity through artisanal interventions circumvents the environmental costs of virgin material production, reinforcing a circular fashion economy [31].

By synthesizing environmental sustainability with cultural preservation, this approach repositions traditional craftsmanship as a critical intervention within the contemporary fashion industry. It presents an alternative to exploitative, resource-intensive production models, offering a transformative blueprint for ethical, heritage-centered fashion innovation.

Table 5 quantifies the STRIE Method’s contributions to multiple Sustainable Development Goals, demonstrating measurable impacts on economic growth, cultural heritage preservation, waste reduction and climate action.

**Table 5:** SDG Alignment and Impact Metrics (2023- 2024).

SDG	Impact Category	Metric	Target Achievement	Source
SDG 8	Economic Growth	Income increase for artisans	45 %	Nair et al. (2023)
SDG 11	Cultural Heritage	Traditional pattern preservation	100 %	Sharma & Bhagat (2023)
SDG 12	Waste Reduction	Pre-consumer waste reduction	20 %	Tripathi et al. [2]
SDG 13	Climate Action	Carbon footprint reduction	79 %	Abrishami et al. [15]

## Potential challenges and mitigation

The potential risks of cultural appropriation necessitate rigorous oversight to ensure ethical engagement with Indigenous craftsmanship. It is imperative that collaborative initiatives prioritize equitable compensation, transparent acknowledgment of artisanship and the preservation of the cultural integrity embedded within traditional embroidery practices. Any form of commercial integration must be guided by ethical frameworks that safeguard the authenticity and agency of Indigenous artisans, preventing the commodification or misrepresentation of their heritage.

Artisans transitioning into large-scale production may initially encounter structural and operational challenges, including navigating logistical complexities and upholding consistent quality standards. To mitigate these barriers, targeted capacity-building programs should be instituted, encompassing specialized training in quality assurance, scalability strategies and sustainable production methodologies. These interventions would not only facilitate artisans’ seamless integration into contemporary fashion ecosystems but also empower them with the skills necessary to maintain artistic autonomy while meeting industrial requirements.

Moreover, market reception of repaired garments featuring traditional Toda embroidery may be met with initial resistance due to prevailing consumer perceptions of upcycled fashion. To counteract such hesitations, strategically crafted storytelling and ethical branding campaigns must foreground the garments’ dual value—sustainability and cultural heritage. By emphasizing the environmental impact reduction, artisanal craftsmanship and social empowerment embedded in these textiles, such campaigns can cultivate demand among ethically conscious consumers who prioritize sustainability and cultural preservation in their purchasing decisions.

## Findings

Sustainability is, at its core, a discourse of equilibrium, quality and respect, as De Castro [7] aptly asserts. Within this paradigm, humanity possesses the agency to reconfigure excess into a practice that resists the fleeting nature of fashion trends. This study introduces an innovative methodological framework that strategically integrates Toda embroidery into garment mending practices, thereby advancing a symbiotic relationship between environmental sustainability and the safeguarding of Indigenous cultural heritage. By leveraging traditional craftsmanship as an intervention for pre-consumer textile waste, this approach not only mitigates the escalating crisis of textile disposability but also fosters sustainable economic pathways for artisan communities.

The incorporation of Toda embroidery within textile repair processes could be demonstrably effective in prolonging garment lifespans while simultaneously augmenting their aesthetic and commercial value through the intricate application of *puhkoor* embroidery. This practice serves as an instrument of economic empowerment for Toda artisans, positioning their craft within the global fashion landscape while reinforcing environmentally sustainable production models. The findings of this study substantiate the claim that the strategic fusion of traditional textile practices with contemporary sustainability initiatives can meaningfully address both ecological imperatives and cultural preservation within the modern fashion industry.

This framework establishes a precedent for a more inclusive, ethical and ecologically responsible trajectory in fashion production. By fostering an intersection between heritage artistry and sustainable innovation, this research redefines the role of Indigenous craftsmanship within contemporary fashion discourses. It serves as a critical call to action for all stakeholders in the fashion ecosystem designers, manufacturers, policymakers and consumers to embrace sustainability in a manner that not only mitigates environmental degradation but also honors and perpetuates cultural traditions as integral to the future of ethical fashion.

## Limitations

Future research could focus on quantitative studies with the Toda community about their concern about developing this method. The implementation faces technical constraints, particularly in the specific nature of repairable defects and time-intensive traditional techniques. Scalability challenges arise from the limited number of skilled Toda artisans and their geographic concentration in the Nilgiris region. Furthermore, developing efficient training programs for artisans, evaluating the long-term economic impact on the Toda community and exploring the adaptability of this approach to other traditional crafts and global markets would provide valuable insights for future development.

## CRediT authorship contribution statement

**Ramya Arivanantham:** Writing – original draft, Methodology, Conceptualization. **Vineeth Radhakrishnan:** Writing – review & editing.

## Declaration of competing interest

The authors declare that they have no known competing financial interests or personal relationships that could have appeared to influence the work reported in this paper.

## Data Availability

No data was used for the research described in the article.

## References

[1] Ahosanul Karim M., Moniruzzaman M., Eanamul Haque Nizam M., Afrin Shammi M., Tanjibul Hasan M. (2021). Transformative adoption of traditional ‘kantha’ embroidery to modern fashion design through ‘khadi’ fabric. Am. J. Art Design.

[2] Tripathi M., Sharma M., Bala S., Thakur V.K., Singh A., Dashora K., Hart P., Gupta V.K. (2024). Recent technologies for transforming textile waste into value-added products: a review. Curr. Res. Biotechnol..

[3] Mohan A., Bali N., Reddy P.L., Balecha M., Dubey M., Sureka A., Khanna P., Maheshwari K., Castle N., Venati H., Bourland M., Sasi R., Rodrigues W., Khubele A., Jariwala P. (2022). Wealth in waste: india’s potential to bring textile waste back into the supply chain. Fashion Good Reports.

[4] Solanki M. (2024). Tripartite Mou to reduce textile waste in India gives hope, but cautious steps needed. Down To Earth.

[5] Allwood J.M., Laursen S.E., de Rodríguez C.M., Bocken N.M.P. (2006). https://www.ifm.eng.cam.ac.uk/uploads/Resources/Other_Reports/UK_textiles.pdf.

[6] Moazzem S., Crossin E., Daver F., Wang L. (2021). Environmental impact of apparel supply chain and textile products. Environ. Dev. Sustain..

[7] De Castro O. (2022). Loved clothes last: how the joy of rewearing and repairing your clothes can be a revolutionary act. Penguin Life.

[8] Woodside A.G., Fine M.B. (2019). Sustainable fashion themes in luxury brand storytelling: the Sustainability fashion research Grid. J. Global Fashion Market..

[9] De Silva R. (2023). Waste isn’t waste until we waste it. Europ. Circ. Econ. Stakeholder Platform.

[10] Yap X.-S., Truffer B. (2019). Shaping selection environments for industrial catch-up and sustainability transitions: a systemic perspective on endogenizing windows of opportunity. Res. Policy..

[11] Singh J., Sung K., Cooper T., West K., Mont O. (2019). Challenges and opportunities for scaling up upcycling businesses – the case of textile and wood upcycling businesses in the UK. Res., Conserv. Recycl..

[12] Sung K., Singh J., Bridgens B., Cooper T. (2021). Lecture Notes in Production Engineering.

[13] Khan A., Tandon P. (2018). Design from discard: a method to reduce uncertainty in upcycling practice. Login.

[14] Stanescu M.D. (2021). State of the art of post-consumer textile waste upcycling to reach the zero waste milestone. Environ. Sci. Pollut. Res..

[15] Abrishami S., Shirali A., Sharples N., Kartal G.E., Macintyre L., Doustdar O. (2024). Textile recycling and recovery: an eco-friendly perspective on textile and garment industries challenges. Textile Res. J..

[16] Igini M. (2024). 10 concerning fast fashion waste statistics. Earth.Org.

[17] Nair K.L., B S., Sunny G. (2022). Cultural documentation and collection development: toda tribes of nilgiris. Chitrolekha J. Art Design.

[18] Sharma G., Bhagat S. (2018). Revival of toda embroidery-needlecraft of nilgiris. Jurnal Sosioteknologi.

[19] Varghese, N., C, N., & Ranka, S. (2020). International Journal of Creative Research Thoughts*.*

[20] Brownlow R., Capuzzi S., Helmer S., Martins L., Normann I., Poulovassilis A. (2015). An ontological approach to creating an Andean weaving knowledge base. J. Comput. Cultur. Herit..

[21] Muthu S.S. (2020).

[22] Sho T. (2022). Kintsugi: Japan’s ancient art of embracing imperfection. BBC News.

[23] GI certificate for toda embroidery formally handed over to tribals. The Hindu. (2013, June 15). https://www.thehindu.com/todays-paper/tp-national/tp-newdelhi/gi-certificate-for-toda-embroidery-formally-handed-over-to-tribals/article4816085.ece

[24] Home: Official website of development commissioner (handicrafts), Ministry of Textiles, Government of India. Home | Official website of Development Commissioner (Handicrafts), Ministry of Textiles, Government of India. 2025. https://handicrafts.nic.in/.

[25] Roos S., Sandin G., Peters G., Bour L.S., Perzon E., Jönsson C., Spak B. (2019). *(pdf) White Paper on textile recycling*. ResearchGate. https://www.researchgate.net/publication/337111016_White_paper_on_textile_recycling.

[26] Environmental sustainability in the fashion industry. Geneva Environment Network. (2024, November 28). https://www.genevaenvironmentnetwork.org/resources/updates/sustainable-fashion/

[27] Dominish E. (2022). Taking climate action: measuring carbon emissions in the garment sector in Asia. Int. Labour Organiz..

[28] Mikucioniene D., Mínguez-García D., Repon Md.R., Milašius R., Priniotakis G., Chronis I., Kiskira K., Hogeboom R., Belda-Anaya R., Díaz-García P. (2024). Understanding and addressing the water footprint in the textile sector: a review. AUTEX Res. J..

[29] Restiani P., Khandelwal A. (2017). https://siwi.org/wp-content/uploads/2017/06/Water-Governance-Mapping-Report-INDIA.pdf.

[30] Premkumar R. (2019). Toda embroidery duplicates threaten local artisans’ livelihood. The Hindu.

[31] Nations United (2023). Take action for the sustainable development goals - United Nations sustainable development. United Nations.

